# The Effect of Autologous Platelet-Rich Gel on the Dynamic Changes of the Matrix Metalloproteinase-2 and Tissue Inhibitor of Metalloproteinase-2 Expression in the Diabetic Chronic Refractory Cutaneous Ulcers

**DOI:** 10.1155/2015/954701

**Published:** 2015-06-28

**Authors:** Lan Li, Dawei Chen, Chun Wang, Guanjian Liu, Xingwu Ran

**Affiliations:** ^1^Diabetic Foot Care Center, Department of Endocrinology and Metabolism, West China Hospital, Sichuan University, Chengdu 610041, China; ^2^Chinese Cochrane Centre, Chinese EBM Centre, West China Hospital, Sichuan University, Chengdu 610041, China

## Abstract

*Aim*. To investigate the dynamic changes on the expression of matrix metalloproteinases (MMPs) and tissue inhibitor of metalloproteinases (TIMPs) in the diabetic chronic refractory cutaneous ulcers after the autologous platelet-rich gel (APG) treatment. *Methods*. The study was developed at the Diabetic Foot Care Centre, West China Hospital. The granulation tissues from the target wounds were taken before and within 15 days after APG application. The expression of MMP-2 and TIMP-2 as well as transforming growth factor-*β*1 (TGF-*β*1) in the granulation tissue was detected by q TR-PCR and IHC. The relationship between the expression level of MMP-2 and TIMP-2 and their ratio and that of TGF-*β*1 was analyzed. *Results*. The expression of MMP-2 (*P* < 0.05) was suppressed, and the expression of TIMP-2 (*P* < 0.05) was promoted, while the ratio of MMP-2/TIMP-2 (*P* < 0.05) was decreased after APG treatments. The expression of TGF-*β*1 had negative correlation with the ratio of MMP-2/TIMP-2 (*P* < 0.05) and positive correlation with the expression of TIMP-2 (*P* < 0.05). *Conclusions*. APG treatment may suppress the expression of MMP-2, promoting that of the TIMP-2 in the diabetic chronic refractory cutaneous wounds. TGF-*β*1 may be related to these effects.

## 1. Introduction

Matrix metalloproteinases (MMPs), a group of zinc-dependent endopeptidases in the degradation of almost all extracellular matrix (ECM), are secreted by keratinocytes and fibroblasts and many other stromal cells. Both collagenases and gelatinases are the most important types, and the latter mainly refer to MMP-2 and MMP-9 and act on collagen type IV-V, gelatin, and so on [1]. The content and activity of MMPs in tissues are regulated by 3 levels of DNA and protein expression, proenzyme activation, and enzyme activity inhibition [2]. Tissue inhibitors of metalloproteinases (TIMPs) take part in the last 2 levels by binding with the fixed sites of the inactive proenzymes or active enzymes and are still influenced by many bioactive substances and cell actions [2, 3]. The known endogenous TIMPs in mammals are classified into 4 types. Of which, TIMP-2 combines with MMP-2 and pro-MMP-2 generally.

It is reported that the dynamic changes on the content and activity of MMP-2 and/or TIMP-2, which are secreted by fibroblasts majorly, play much essential parts in the normal healings, especially during the midterm and later phases, including accelerating revascularization, granulation tissues regeneration as well as the connective tissues reformation, and safeguarding the normal dermis to some extent [4]. The peak of their content overlaps that of granulation tissues regeneration velocity and the high level maintains for 14 days or longer [4].

Owning to the decreased content and/or activity of growth factors and the disturbed balance of MMPs/TIMPs system, which results in excessive solvent activity and then reduced content or damaged structure of the growth factors and other ECMs locally, the diabetic cutaneous ulcers are always poorly healed. MMP-2 is found to be excessively generated while TIMP-2 is deficiently secreted in diabetic chronic wounds, and the pathologic imbalance may bring about retarded progress of tissue regeneration and revascularization [5].

Within the recent 10 years, the intelligent efficacy of autologous platelet-rich gel (APG) on refractory wounds has been reported [6, 7]. In our preliminary RCT, the cumulative rate of ulcer healing was 95.7% in the APG group versus 56.5% in the standard treatment group (*P* = 0.002); the total effective rate in APG versus standard treatment group was 100.0% versus 73.9% (*P* = 0.009) [8]. The healing-promoting mechanism is recognized including upregulating the content of many growth factors and releasing antibacterial peptides [9–11]. More recently, some platelet-derived wound healing factors (PDWHFs) were reported to influence the MMP-2/TIMP-2 and other pathways in MMPs/TIMPs system in cellular and animal studies [1, 12] and our preliminary clinical experiment further provides some positive evidence about APG treatment on another pathway of MMP-1, MMP-9, and TIMP-1 [13]. However, the data on the effects of APG treatment on the MMP-2/TIMP-2 pathway in diabetic chronic wounds were relatively insufficient.

Furthermore, TGF-*β*1 has been reported in some basic researches to inhibit the generation of MMP-2 by depressing its genetic transcription and enhance that of TIMP-2 meanwhile [1]. In our preliminary clinical study, the local concentration of TGF-*β*1 increases 2-3-fold after APG treatment [9]. So TGF-*β*1 is properly suspected to join in the regulation of APG treatment on MMP-2 and TIMP-2. But so far, no direct evidence has ensured the hypothesis.

Therefore, this study progressively and creatively aimed to investigate the dynamic changes on the expression of MMP-2 and TIMP-2 in the diabetic chronic refractory cutaneous ulcers after the APG treatment as well as their correlation with TGF-*β*1.

## 2. Materials and Methods

### 2.1. Study Design and Population

The study aimed to investigate the dynamic changes on the expression of MMP-2 and TIMP-2 as well as the ratio of MMP-2/TIMP-2 in the diabetic chronic refractory cutaneous ulcers after the APG treatment and then calculate their correlation with TGF-*β*1. It was carried out at the Diabetic Foot Care Center, West China Hospital, Sichuan University (Sichuan, China) between April 1, 2012, and December 31, 2012. All eligible patients experienced 12-week observation and therapies. The study protocol, informed consent form, and other study related documents were reviewed and approved by the Ethics Committee of West China Hospital.

All eligible patients, screened according to the following inclusion and exclusion criteria, signed the informed consent and prescribed with APG treatment. Those, whose granulation tissues from the target wounds were taken at every observation point before and within 15 days after the first APG application, were taken into analysis. Diabetic patients over 18 years of age, with at least one cutaneous ulcer (area of 1~60 cm^2^) or sinus (volume of 1 cm^3^ or more) lasting no less than 4 weeks and not meeting an area size reduction of 10–15% per week after at least two-week standard treatments for ulcers, participated in the study [9].

### 2.2. Treatment Procedures

During the prerecruitment period, all of the participants received systemic therapies and standard care for the cutaneous ulcers. The former consisted of intensive insulin therapy, anti-infection, nerve-trophic and circulation-improving therapies, and so on. The latter composed of topical washing, cleaning, draining, and debridement to remove callous, necrotic tissue and sequestra, as well as dressing changes. Proper limb immobilization and weight offloading were prescribed. Within the treatment period, above-mentioned systemic therapies continued. Participants were prescribed with a topical application of APG upon the wound beds before administration of Suile wound dressing (Hedonist Biochemical Technologies Co., Ltd., USA) and occlusive bandages at baseline. The procedure of APG preparation was described in detail by Yuan et al. [14]: following centrifugation at 313 ×g for 4 minutes, erythrocyte concentrate was removed. PRP and PPP were prepared by centrifugation (1252 ×g) for 6 minutes from the remaining plasma. Thrombin and calcium gluconate were added to PRP, and the gel-like mixture is called APG. Then, Suile dressing and occlusive bandages were maintained for 3 days and then changed at 3-day intervals.

### 2.3. Evaluations and Endpoints

Wound evaluations were taken along with dressing and bandages changing every 3 days. The granulation tissues within a 5 mm range from the centre of wound bed were also biopsied along with dressing changing and then maintained in the paraformaldehyde or liquid nitrogen for measuring until the area of less than 0.5 cm^2^ or volume of less than 0.5 cm^3^.

The expression of each target mRNA in the granulation tissue was detected semiquantitatively by quantitative reverse transcription polymerase chain reaction (q TR-PCR). The total RNA was extracted by the Trizol (Roche Dia) method. The high capacity cDNA synthesis kit (TaKaRa Bio Inc.) was applied to carry out the reverse-transcription and generate cDNA. The q RT-PCR (Chromo4, Bio Pad) was carried out by SYBR Green (Roche Dia) method. The primers were synthesized according to related articles by Invitrogen Life Technology Co., Ltd. (Shanghai) (Table 1).

The expression of each target protein in the granulation tissue was locatively and semiquantitatively detected by immunohistochemistry technique (IHC). Immunohistochemistry staining was mediated by ChemMate Envision+HRP/DAB. Primary antibodies were unique: mouse monoclonal antibody of MMP-2 (Abcam), dilution ratio of 1 : 300; rabbit polyclonal antibody of TGF-*β*1 (Abcam), dilution ratio of 1 : 400; rabbit polyclonal antibody of TIMP-2 (Abcam), dilution ratio of 1 : 100. Secondary antibodies were from common kit for ChemMate Envision+HRP/DAB (Gene Technology Co., Ltd., Shanghai).

Each section was evaluated for the area and density of staining by microscopic examination (×200; BX51; Olympus, Tokyo, Japan). Two noncontiguous microscopic areas were randomly selected and photographed with a digital camera (DP72; Olympus, Tokyo, Japan). For each selected area, a digital image (1360 × 1024 pixels) was captured and stored as high-resolution image file. To measure the extent of immunoreactivity, computer-aided image analysis software (Image-Pro Plus, Media Cybernetics) was introduced to discriminate the immunostained area, calculate the integrated optical density and immunoreactive area, and count the mean density.

According to our preliminary study, the promoting action of APG on the healing of diabetic chronic ulcer was considered functioning within 15 days after the APG treatment [6]. So, repeated APG treatments were performed every two weeks or 15 days, if necessary. And we analyzed the data within 15 days after the APG treatment.

### 2.4. Statistical Analysis

Numerical data with normal distribution was represented by mean ± standard deviations (SDs); otherwise, it was represented by median, interquartile range (IQR). Repeated measurement data were tested by general linear model analysis (GLM) and the correlation by Pearson correlation analysis. All tests were two-sided, and *P* value of 0.05 was considered significant.

## 3. Results and Discussion

### 3.1. Results

#### 3.1.1. Epidemiologic Data

A total 51 diabetic inpatients were screened in our centre during this trial and 25 patients were enrolled and prescribed with the APG treatment. Nineteen eligible patients, whose granulation tissues from the target wounds were taken at every observation point before and within 15 days after the first APG application, were taken into analysis, while the rest 6 were excluded for healing too fast to reach the ulcer areas of less than 1 cm² before the d15 after the first APG treatment (*n* = 3) or being intolerant to painfulness relevant to the operations (*n* = 3). The 19 patients were aged 43.5 ± 10.1 years, with the diabetic and cutaneous wound duration of 12, 3~16.5 years and 4, 4~14 weeks, respectively. The majority (17, 89.47%) of the participants were male. Each participant maintained normal blood glucose, pressure, and lipid, as well as hepatic and renal functions.

#### 3.1.2. Dynamic Changes on the mRNA Expression of MMP-2, TIMP-2, and TGF-*β*1 as well as the Ratio of MMP-2/TIMP-2 by qRT-PCR before and after the APG Application

**Figure 1:**
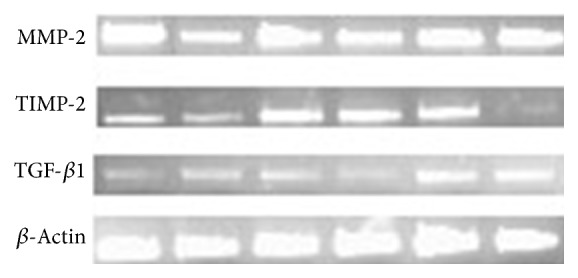
Electrophoretic bands of the q RT-PCR products before and within 15 days after the APG application. Note: the electrophoretic bands of the q RT-PCR products from left to right represent the expression of each target mRNA from the d0, d3, d6, d9, d12, and the d15 one by one.

**Figure 2:**
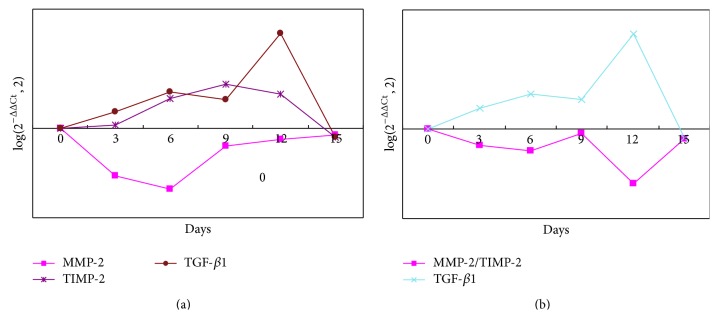
Dynamic changes of the mRNA expression of MMP-2, TIMP-2, and TGF-*β*1 as well as the ratio of MMP-2/TIMP-2 in the granulation tissue of wounds before and within 15 days after the APG application.

Figure 1 showed the electrophoretic bands of the qRT-PCR products of MMP-2, TIMP-2, TGF-*β*1, and *β*-actin and roughly the expression of these genes before and after APG treatment. The mRNA expression of MMP-2 in the granulation tissues decreased within 12 days after the APG application (*P* = 0.007), that of TIMP-2 increased progressively with the peak appearing during the d9–d12 (*P* < 0.0001), and that of TGF-*β*1 increased with the peak at the d12 (*P* = 0.004) (Figure 2(a)). Meanwhile, the ratio of MMP-2/TIMP-2 decreased with the trough at the d12 after the APG application (*P* < 0.0001) (Figure 2(b)).

#### 3.1.3. The Correlation between the mRNA Expression of TGF-*β*1 and That of MMP-2, TIMP-2 as well as the Ratio of MMP-2/TIMP-2

The mRNA expression of TGF-*β*1 had positive correlation with TIMP-2 (*P* = 0.028, *γ* = 0.680), no correlation with MMP-2 (*P* > 0.05, *γ* = −0.071), and meanwhile negative correlation with the ratio of MMP-2/TIMP-2 (*P* = 0.032, *γ* = −0.809) within 15 days after the APG application.

#### 3.1.4. Dynamic Changes on the Protein Expression of MMP-2, TIMP-2, and TGF-*β*1 by IHC before and after the APG Application

**Figure 3:**
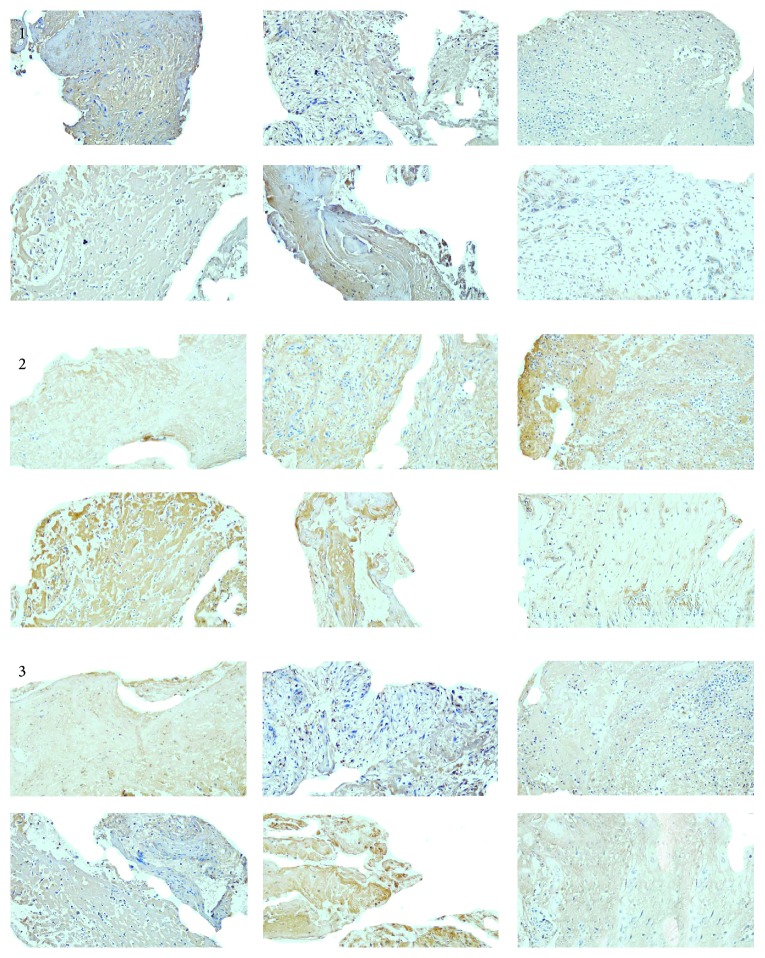
Dynamic changes on the expression of each target protein in granulation tissue before and within 15 days after the APG application. Note: the images captured from sections present the dynamic changes of each target protein within 15 days after the first APG application. The immunoreactants in the images from number 1 to 3 were MMP-2, TIMP-2, and TGF-*β*1 one by one. Each number of images includes 6 parts, which were taken at the d0, d3, d6, d9, d12, and d15 from left to right. The expression of MMP-2 was suppressed after APG treatment and that of TIMP-2 was promoted by APG treatment, and levels at each observation point were higher than baseline before the d12, with the peak appearing at about the d9–12. TGF-*β*1 was promoted also, and the peak came out at the d12.

**Figure 4:**
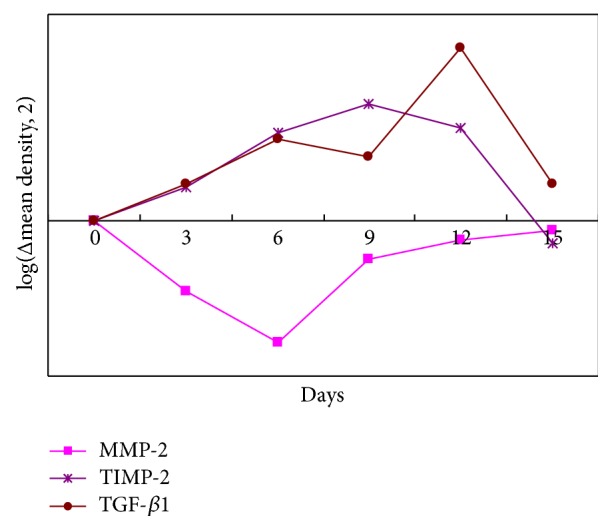
Dynamic changes of the protein expression of MMP-2, TIMP-2, and TGF-*β*1 before and within 15 days after the APG application. Dynamic changes of the MMP-2, TIMP-2, and TGF-*β*1 before and within 15 days after the first APG application.

Different shades of brown yellow granules were observed in the immunohistochemical staining sections. It is explained that each target protein expressed differently in ECM and the plasma of positive cells (Figure 3). The dynamic changes of the protein expression by IHC and photodensitometry paralleled with the mRNA expression by q RT-PCR (Table 2, Figure 4), and the differences were all statistically significant after the APG application (MMP-2, *P* = 0.001; TIMP-2, *P* < 0.0001; TGF-*β*1, *P* = 0.0134).

#### 3.1.5. The Correlation between the Protein Expression of TGF-*β*1 and That of MMP-2 as well as TIMP-2

The protein expression of TGF-*β*1 had positive correlation with that of TIMP-2 (*P* = 0.041, *γ* = 0.660) and no correlation with MMP-2 (*P* > 0.05, *γ* = −0.063) within 15 days after the APG application.

### 3.2. Discussion

In this study, the mRNA expression of MMP-2, TIMP-2, and TGF-*β*1 from qRT-PCR technique is similar to the protein expression from IHC. The (mRNA and protein) expression of MMP-2 decreases and that of TIMP-2 increases after APG treatment, and the expression of TGF-*β*1 has positive correlation with that of TIMP-2. Meanwhile, the ratio of MMP-2/TIMP-2, from q RT-PCR, decreases after the APG application and has negative correlation (trend) with the expression of TGF-*β*1. To sum up, we conclude that APG treatment promotes the expression of TIMP-2, inhibits that of MMP-2, and decreases the ratio of MMP-2/TIMP-2 in diabetic refractory wounds, and TGF-*β*1 might be related to these effects.

There were some studies referring to the effects of APRP or other PDWHFs on the expression of MMP-2 and/or TIMP-2, but resulting differently. Cáceres et al. [15] reported that APRP stimulated the expression of TGF-*β*1 and TIMP-2 in gingival fibroblasts; Shin and Oh [16] found that PRP promoted the mRNA expression of MMP-2 and MMP-9 in the wound of OLETF (Otsuka Long-Evans Tokushima fatty rats). Furthermore, Axelrad et al. [17] showed that platelet-activating factors (PAF) excited the mRNA expression of TIMP-2 in the human umbilical vein endothelial cells (HUVEC), but not that of MMP-2. In short, the studies on the MMP-2/TIMP-2 pathway were only a few and almost applying animal and cellular models with simple reaction environment and/or intervenes, while those on the chronic refractory wounds upon APG/APRP treatment, especially clinical trials, were rare. This is before we implemented a clinical trial and investigated the dynamic changes on another pathway (MMP-1 and MMP-9/TIMP-1) of MMPs/TIMPs but not MMP-2/TIMP-2 [13]. So, this study was creatively directed against the MMP-2/TIMP-2 pathway and found a decreased ratio of MMP-2/TIMP-2 upon APG treatment, another potential healing-promoting mechanism of it.

Many basic researches have reported the effects of TGF-*β*1 on MMPs/TIMPs system and showed its inhibition on MMP-2 expression and promotion to TIMP-2 [1]. This study shows that TGF-*β*1 might participate in inhibiting MMP-2 expression (negative correlation or trend) while promoting TIMP-2 and further decrease the ratio of MMP-2/TIMP-2, which coincides with the previous basic finds. It ensures the previous hypothesis of TGF-*β*1 mediating the action of APG and provided clinical data on the effects of TGF-*β*1 on the pathway of MMP-2.

Previously, no studies (especially the clinical) aimed to show the effects of the APG/PRP treatment on the dynamic changes of MMP-2/TIMP-2 pathway. We make up the deficiency in the dependent field and initiate further exploring. However, we do not set up a controlled group to be compared with and do not analyze a relatively small sample size; so further work is necessary.

Turning the healing microenvironment of chronic wounds into the acute or acute-like ones, through positive local treatment, is a key point to overcome the challenge of chronic refractory wound. Balancing the ratio of MMPs/TIMPs is an important way to improve the healing condition and accelerate the healing velocity for diabetic wounds. MMP-2/TIMP-2 pathway is a crucial ingredient of MMPs system acting in, especially the midterm and later phases of healing, and necessary for the granulation tissues regeneration and the connective tissues reformation and so on. Actually, it is worthwhile to focus on not only the APG treatment but also all the new local therapies.

## 4. Conclusions 

APG treatment may suppress the expression of MMP-2 and promote that of TIMP-2 in the diabetic chronic refractory cutaneous wounds and furthermore decrease the ratio of the MMP-2/TIMP-2. TGF-*β*1 may be related to these effects.

## Figures and Tables

**Table 1:** Primer sequences used for the amplification of each target DNA.

Primer	Sequence
TGF-*β*1 forward	TGGCGATACCTCAGCAACC
TGF-*β*1 reverse	CTCGTGGATCCACTTCCAG
MMP-2 forward	GGCCCTGTCACTCCTGAGAT
MMP-2 reverse	GGCATCCAGGTTATCGGGGA
TIMP-2 forward	GGCGTTTTGCAATGCAGATGTAG
TIMP-2 reverse	CACAGGAGCCGTCACTTCTCTTG
*β*-Actin forward	GCGAGAAGATGACCCAGATCATGTT
*β*-Actin reverse	GCTTCTCCTTAATGTCACGCACGAT

**Table 2:** Dynamic changes of the mean density (MD) of the MMP-2, TIMP-2, and TGF-*β*1 in granulation tissue before and within 15 days after the APG application (IHC).

	MMP-2	TIMP-2	TGF-*β*1
Days after APG application			
D0	0.0210 ± 0.0135	0.00282 ± 0.00979	0.00268 ± 0.00192
D3	0.0082 ± 0.0085	0.00404 ± 0.00371	0.00443 ± 0.00850
D6	0.0042 ± 0.0022	0.00931 ± 0.00437	0.00797 ± 0.00118
D9	0.0125 ± 0.0121	0.01368 ± 0.02060	0.00637 ± 0.00731
D12	0.0163 ± 0.0119	0.00991 ± 0.00870	0.00276 ± 0.0672
D15	0.00261 ± 0.00171	0.00210 ± 0.00297	0.00440 ± 0.00291
